# Effects of vitamin D supplementation on metabolic and endocrine parameters in PCOS: a randomized-controlled trial

**DOI:** 10.1007/s00394-018-1760-8

**Published:** 2018-06-26

**Authors:** Christian Trummer, Verena Schwetz, Martina Kollmann, Monika Wölfler, Julia Münzker, Thomas R. Pieber, Stefan Pilz, Annemieke C. Heijboer, Barbara Obermayer-Pietsch, Elisabeth Lerchbaum

**Affiliations:** 1 Division of Endocrinology and Diabetology, Department of Internal Medicine, Medical University of Graz, Auenbruggerplatz 15, 8036 Graz, Austria; 2 Department of Obstetrics and Gynecology, Medical University of Graz, Auenbruggerplatz 14, 8036 Graz, Austria; 3 Department of Medicine, Integrated Research and Treatment Centre for Adiposity Diseases, Leipzig University, Philipp-Rosenthal-Straße 27, 04103 Leipzig, Germany; 4 Endocrine Laboratory, Department of Clinical Chemistry, VU University Medical Center, De Boelelaan 1117, 1081 HV Amsterdam, The Netherlands; 5 Laboratory of Endocrinology, Academic Medical Center, Meibergdreef 9, 1105 AZ Amsterdam, The Netherlands

**Keywords:** Vitamin D supplementation, PCOS, RCT, Insulin resistance, Glucose sensitivity

## Abstract

**Purpose:**

Vitamin D status may be associated with insulin resistance and other key features of polycystic ovary syndrome (PCOS), but data from preliminary randomized controlled trials (RCTs) are conflicting. Therefore, we aimed to investigate the effects of vitamin D supplementation on plasma glucose area under the curve (AUCgluc, primary outcome measure) and on other metabolic and endocrine parameters (secondary outcome measures).

**Methods:**

This study was a single-center, double-blind, randomized placebo-controlled trial conducted between December 2011 and July 2017 at the Medical University of Graz, Austria. One-hundred and eighty women with PCOS and 25-hydroxyvitamin D [25(OH)D] concentrations < 75 nmol/L were randomized in a 2:1 ratio to either receive 20,000 IU of cholecalciferol weekly or placebo over 24 weeks. Primary outcome was the between-group difference in AUCgluc at study end while adjusting for baseline values.

**Results:**

In total, 123 participants completed the study [age 25.9 ± 4.7 years; BMI 27.5 ± 7.3 kg/m^2^; baseline 25(OH)D 48.8 ± 16.9 nmol/L, baseline fasting glucose 84 ± 8 mg/dL]. Vitamin D supplementation lead to a significant increase in 25(OH)D [mean treatment effect 33.4 nmol/L; 95% confidence interval (CI) 24.5 to 42.2; *p* < 0.001] but had no significant effect on AUCgluc (mean treatment effect − 9.19; 95% CI − 21.40 to 3.02; *p* = 0.139). Regarding secondary outcome measures, we observed a significant decrease in plasma glucose at 60 min during oral glucose tolerance test (mean treatment effect − 10.2 mg/dL; 95% CI − 20.2 to − 0.3; *p* = 0.045).

**Conclusions:**

Vitamin D supplementation had no significant effect on metabolic and endocrine parameters in PCOS with the exception of a reduced plasma glucose during OGTT.

**Electronic supplementary material:**

The online version of this article (10.1007/s00394-018-1760-8) contains supplementary material, which is available to authorized users.

## Introduction

Polycystic ovary syndrome (PCOS) is the most common endocrine disorder among women of reproductive age [1]. PCOS is a very heterogeneous condition with potential implications for reproductive, metabolic, and psychological features [2].

While vitamin D deficiency itself is very common in the general population, it is even more prevalent in PCOS patients [3–5]. As vitamin D status appears to be closely linked to insulin resistance, one of the key features of the PCOS phenotype, vitamin D supplementation might improve insulin sensitivity [6–8]. Vitamin D may lead to a suppression of proinflammatory cytokines and increase the expression of the insulin receptor, thereby enhancing insulin synthesis and release [4, 9]. Insulin resistance is associated with an increased risk of several metabolic disturbances, including type 2 diabetes mellitus and cardiovascular disease [10, 11]. Furthermore, metabolic disturbances in PCOS are related to ovarian physiology [12], leading to the assumption that vitamin D supplementation may also have a positive impact on menstrual frequency and serum androgen levels. This is underscored by the ubiquitous expression of the vitamin D receptor (VDR) within the female reproduction system [13–15].

The current treatment options for PCOS mainly consist of lifestyle intervention, hormonal contraceptives and insulin sensitizers [16]. Considering the high-prevalence of vitamin D deficiency in PCOS, vitamin D supplementation could be a simple and low-risk add-on to these therapies if its positive effects on metabolic and endocrine features were proven to be true. Thus, several studies in the recent past including some randomized-controlled trials (RCTs) aimed to evaluate the effects of vitamin D supplementation on characteristics of the PCOS phenotype. However, these studies have mostly yielded mixed results and were, at least in part, limited due to their varying study design or the small number of study participants [17].

The aim of the present study was to investigate the effect of vitamin D supplementation in 180 women with PCOS. Our main study aim was to evaluate whether vitamin D supplementation as compared to placebo has an effect on plasma glucose area under the curve (AUCgluc) as a measure of glucose excursion. As secondary outcomes, we investigated the effects of vitamin D supplementation on several other metabolic and endocrine parameters including serum testosterone levels and menstrual frequency.

## Materials and methods

### Study design

This study was a single-center, randomized, double-blind, placebo-controlled trial conducted at the Medical University of Graz, Austria. The trial was designed to investigate the effects of vitamin D supplementation over 24 weeks on metabolic and endocrine parameters in women with PCOS. To investigate possible short-time effects of vitamin D supplementation, an additional follow-up study visit was scheduled 12 weeks after inclusion into the study. The design, conduction, and publication of this trial adhere to the Consolidated Standards of Reporting Trials (CONSORT) 2010 statement [18]. The trial was registered at clinicaltrials.gov (ClinicalTrials.gov Identifier NCT01721915) and at http://www.clinicaltrialregister.eu (EudraCT number, 2011-000994-30). The study protocol was approved by the ethics committee of the Medical University of Graz.

### Study subjects

Eligible study subjects were premenopausal women aged ≥ 18 years with PCOS and 25-hydroxyvitamin D [25(OH)D] serum concentrations < 75 nmol/L (divide by 2.496 to convert nmol/L to ng/mL). A threshold of < 75 nmol/L was chosen to define vitamin D insufficiency based on existing guidelines of the Endocrine Society [19]. Diagnosis of PCOS was established according to the Rotterdam criteria [20], if two out of the following three characteristics were met: oligo-/anovulation, clinical and/or biochemical signs of hyperandrogenism, and/or polycystic ovaries (diagnosed by ultrasound). Disorders with similar clinical features, e.g., congenital adrenal hyperplasia, Cushing’s syndrome, or androgen-secreting tumors, were excluded before the diagnosis of PCOS was made. Exclusion criteria were hypercalcemia (defined as plasma calcium concentrations > 2.65 mmol/L), hormonal contraception within 3 months prior to study inclusion, use of insulin-sensitizing drugs (i.e., metformin, incretin mimetic drugs, thiazolidinedione, sulfonylurea) within 6 months prior to study inclusion, use of lipid-lowering drugs or other drugs affecting insulin sensitivity or serum androgens (e.g., niacin, corticosteroids, beta-blockers, calcium channel blockers, thiazide diuretics), prevalent type 2 diabetes mellitus, any other disorder apart from PCOS associated with androgen excess and/or menstrual irregularity, and regular vitamin D supplementation within 3 months prior to study inclusion.

Study participants were recruited from the outpatient clinic of the Division of Endocrinology and Diabetology, Department of Internal Medicine, Medical University of Graz, Austria. Patients were informed about the trial by conversation during their routine visit in the outpatient clinic, by telephone call, or by written information posted in the outpatient clinic. All study participants gave written informed consent prior to any study related procedures.

### Intervention

Subjects were allocated in a 2:1 ratio to receive either vitamin D or placebo by a computer-generated randomization list using a web-based software (http://www.randomizer.at) with good clinical practice compliance as confirmed by the Austrian Agency for Health and Food Safety (AGES). Since we further aimed to analyze the response to vitamin D supplementation according to genotype profile (also see subsection Secondary outcome measures), we randomized patients 2:1 (vitamin D:placebo) to increase the sample size in the vitamin D treatment group.

Study medication and placebo were filled into numbered bottles according to the generated randomization list. Study participants in the intervention group received 20,000 IU of cholecalciferol weekly equaling 50 oily drops per week (Oleovit D3-drops; Fresenius Kabi Austria GmbH, Linz, Austria) for 24 weeks, while participants in the placebo group received 50 oily drops without cholecalciferol per week for 24 weeks. Placebo and study medication could not be distinguished by look, smell, or taste. All investigators involved in enrollment of participants, collection of data, and assignment of intervention were masked to participant allocation. To verify and improve participant compliance, all study subjects were asked to return the empty study medication bottles at the final study visit after 24 weeks.

### Primary outcome measure

The primary outcome measure was the between-group difference in AUCgluc during oral glucose tolerance test (OGTT) after 24 weeks.

### Secondary outcome measures

Secondary outcome measures were the between-group differences in insulin resistance [assessed by homeostatic model assessment-insulin resistance (HOMA-IR)], total cholesterol (TC), glycated hemoglobin (HbA1c), total (TT) and free testosterone (FT), menstrual frequency, insulin sensitivity [assessed by quantitative insulin-sensitivity check index (QUICKI)], and triglycerides after 24 weeks. As described above, primary and secondary outcome measures were additionally assessed at 12 weeks to detect possible short-term effects of vitamin D treatment.

As pre-specified, another secondary outcome of the underlying study was to evaluate the association of changes in metabolic and endocrine parameters with vitamin D-related gene variants. However, for reasons of legibility and space, we decided not to include these analyses in the current manuscript.

### Procedures

Physical examinations, blood samplings, and patient interviews were conducted at each study visit between 8.00 and 9.00 a.m. after an overnight fast of at least 12 h. Both 25(OH)D and TT were initially measured by immunoassays to evaluate inclusion criteria and establish the diagnosis of PCOS. Remaining blood samples were stored at − 80 °C until batch analysis. Serum concentrations of 25(OH)D and TT were additionally measured by well-standardized isotope-dilution liquid chromatography tandem mass spectrometry (ID-LC-MS/MS) in 2017 at the VU University Medical Center, Amsterdam, the Netherlands [21–23]. Statistical analyses for this manuscript were performed with ID-LC-MS/MS measurements of 25(OH)D and TT. FT was calculated from TT (measured by ID-LC-MS/MS), sex-hormone binding globulin (SHBG), and albumin as published by Vermeulen [24]. The free androgen index (FAI) was calculated as TT (measured by ID-LC-MS/MS) (nmol/L)/SHBG (nmol/L) × 100.

At the screening visit, eligible study participants were randomized and received the study medication as well as appointments for follow-up visits after 12 and 24 weeks, respectively. Additionally, printed menstrual calendars were handed out and participants were asked to document menstrual frequency and duration during study participation. Menstrual calendars were returned at the final study visit to evaluate changes in menstrual frequency.

At each study visit, participants underwent a fasting 75 g OGTT. Blood samples were drawn at baseline and after 30, 60, and 120 min for measurement of glucose and insulin. AUCgluc was calculated according to the trapezoidal method. To estimate insulin resistance, HOMA-IR was calculated as fasting plasma insulin (µU/mL) × fasting plasma glucose (mg/dL)/405. As a measure for insulin sensitivity, QUICKI was calculated as 1/log fasting insulin (µU/mL) + log fasting glucose (mg/dL) [25]. Further methods are described in the supplemental Materials and Methods section.

Normal ranges of biochemical and anthropometric parameters (where available) are summarized in Supplemental Table 1.

### Statistical analysis

Sample size calculation was based on the results of a pilot study conducted at our department [26], reporting a reduction of AUCgluc from 115 ± 17 at baseline to 103 ± 18 after vitamin D supplementation over 24 weeks. Therefore, a sample size of 92 participants was calculated to detect a treatment difference at a two-sided 0.05 significance level with a probability of 90%, if the true difference between treatments is 12 with a standard deviation (SD) of 17. As the drop-out rate turned out to be higher than expected when recruitment was completed, the number of enrolled study participants was increased from 150 to 180 to ensure an adequate power to detect differences regarding the primary outcome measure.

Continuous data with normal distribution are presented as means with SD, while continuous data following a skewed distribution are shown as medians with interquartile range. Categorical data are presented as percentages. Data distribution was analyzed by descriptive statistics and Kolmogorov–Smirnov test. Unpaired Student’s *t* test, Mann–Whitney *U* test, *Χ*^2^ test, and Fisher’s exact test were used for baseline comparisons between the vitamin D and placebo group, depending on variable type and data distribution. According to the patient interview and the returned menstrual calendars, menstrual frequencies both before study enrollment and during study participation were categorized as follows: normal menstrual frequency (menstrual cycle duration 21–35 days), oligomenorrhea (menstrual cycle duration > 35 days), hypermenorrhea (menstrual cycle duration < 21 days), or amenorrhea (absence of menses for more than 6 months). To investigate the effect of vitamin D supplementation in participants with particularly low 25(OH)D concentrations, we performed subgroup analyses in patients with a baseline 25(OH)D serum level < 50 and < 40 nmol/L, as these concentrations are considered to cover the need of 97.5 and 50% of the population, respectively [27]. Analyses of outcome variables were performed according to the intention-to-treat principle with no data imputation and inclusion of all participants with available baseline and follow-up values of the respective outcome variable. ANCOVA with adjustments for baseline values was used to test for differences in continuous outcome variables between the treatment and the placebo group at the respective follow-up visit. Skewed variables were log(*e*) transformed before they were used in statistical analyses requiring parametric distribution. *X*^2^ test was used to test for differences between the groups regarding improvement of menstrual frequency. An improvement of menstrual frequency was defined as a transition from amenorrhea to oligomenorrhea/hypermenorrhea or as a transition from either amenorrhea or oligomenorrhea/hypermenorrhea to normal menstrual frequency. A *p* value < 0.05 was considered statistically significant. All statistical operations were performed with SPSS version 23 software (SPSS Inc., Chicago, IL, USA).

## Results

**Fig. 1:**
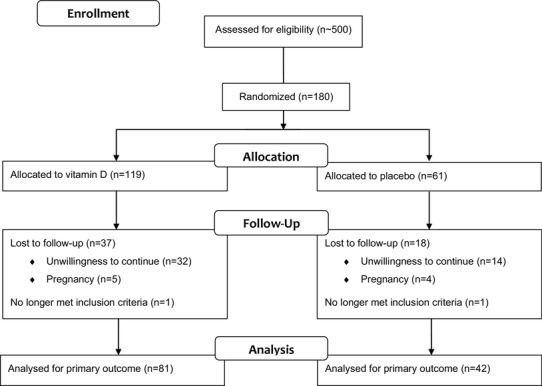
Participant flow-chart

Approximately 500 patients who underwent investigation for PCOS were screened, 180 patients matching the inclusion criteria were recruited. The first patient was randomized in December 2011, the last follow-up visit was performed in July 2017. A participant flow-chart is shown in Fig. 1. Despite rigorous monitoring, two patients had to be excluded from the study after randomization, since both no longer matched the PCOS inclusion criteria by developing a regular menses shortly after the screening visit. However, adhering to the intention-to-treat principle, we did not exclude these study participants from the final analyses.

Baseline characteristics of all randomized participants are shown in Table 1. Participants in the vitamin D group were significantly younger and had higher serum glucose concentrations at 60 min during OGTT when compared to the placebo group. All other characteristics did not show any significant differences between the groups at baseline (Table 1).

**Table 1:** Baseline characteristics of all randomized study participants

Characteristics	All (*n* = 180)	Vitamin D (*n* = 119)	Placebo (*n* = 61)	*p* value
Age (years)	26.0 ± 5.0	25.4 ± 4.6	27.2 ± 5.5	0.022
Body-mass index (kg/m^2^)	27.6 ± 7.5	27.3 ± 7.4	28.3 ± 7.8	0.453
Waist circumference (cm)	89.0 (78.3–104.0)	87.0 (77.0–104.0)	93.0 (82.0–104.5)	0.210
Hip circumference (cm)	102.0 (94.1–116.8)	101.0 (94.0–115.0)	105.0 (95.5–118.5)	0.378
WHR (cm/cm)	0.87 ± 0.10	0.86 ± 0.08	0.88 ± 0.12	0.245
Systolic BP (mmHg)	122 ± 13	122 ± 13	122 ± 13	0.803
Diastolic BP (mmHg)	81 ± 10	81 ± 10	82 ± 10	0.214
Fasting glucose (mg/dL)	84 ± 8	84 ± 8	84 ± 7	0.859
OGTT glucose 30 min (mg/dL)	130 ± 26	131 ± 27	126 ± 23	0.247
OGTT glucose 60 min (mg/dL)	117 ± 37	121 ± 39	109 ± 32	0.044
OGTT glucose 120 min (mg/dL)	97 ± 25	99 ± 24	93 ± 25	0.150
AUCgluc	222.09 ± 44.5	226.71 ± 46.12	213.07 ± 40.03	0.051
Fasting insulin (mU/L)	10.1 (5.8–16.1)	10.3 (5.7–16.8)	9.9 (6.3–13.6)	0.845
HbA1c (mmol/mol)	34 (31–35)	33 (31–35)	34 (32–35)	0.683
HOMA-IR	2.07 (1.18–3.47)	2.10 (1.12–3.59)	2.04 (1.31–2.80)	0.825
QUICKI	0.342 (0.318–0.373)	0.341 (0.316–0.376)	0.343 (0.327–0.367)	0.825
Triglycerides (mg/dL)	68 (50–94)	66 (50–92)	72 (50–109)	0.388
Total cholesterol (mg/dL)	175 (154–197)	173 (157–191)	176 (149–203)	0.565
HDL-cholesterol (mg/dL)	64 ± 19	63 ± 19	65 ± 20	0.720
LDL-cholesterol (mg/dL)	96 ± 33	94 ± 28	100 ± 41	0.283
CRP (mg/L)	1.1 (0.0–3.6)	1.4 (0.0–3.9)	0.8 (0.0–3.3)	0.350
25(OH)D (nmol/L)	50.4 ± 19.0	50.7 ± 19.5	49.9 ± 18.3	0.798
PTH (pg/mL)	41.6 (34.1–52.5)	41.9 (34.4–53.8)	40.2 (33.0–51.4)	0.595
Plasma calcium (mmol/L)	2.36 ± 0.08	2.36 ± 0.08	2.36 ± 0.07	0.944
Total testosterone (nmol/L)	1.50 (1.10–1.95)	1.50 (1.10–2.10)	1.40 (1.10–1.80)	0.315
Free testosterone (nmol/L)	0.021 (0.015–0.032)	0.021 (0.016–0.032)	0.018 (0.013–0.032)	0.221
FAI	3.14 (2.18–5.26)	3.33 (2.26–5.29)	2.53 (2.04–5.15)	0.223
Androstendione (ng/mL)	3.36 (2.51–4.44)	3.41 (2.43–4.46)	3.32 (2.58–4.41)	0.850
DHEAS (µg/mL)	1.90 (1.34–2.78)	1.94 (1.34–2.70)	1.90 (1.42–2.79)	0.897
Estradiol (pg/mL)	60.6 (44.6–96.0)	59.1 (42.3–91.2)	64.0 (49.5–118.5)	0.164
FSH (mU/mL)	5.97 ± 2.41	5.94 ± 2.33	6.04 ± 2.59	0.783
LH (mU/mL)	9.56 ± 5.60	9.79 ± 5.87	9.11 ± 5.05	0.437
Menstrual irregularity (%)	89.4	89.9	88.5	0.801
Oligomenorrhea (%)	71.7	73.1	68.9	0.549
Hypermenorrhea (%)	2.2	1.7	3.3	0.605
Amenorrhea (%)	15.6	15.1	16.4	0.824

Data are shown as means with standard deviation, medians and interquartile range, or as percentages, as appropriate. Comparisons of baseline characteristics between the vitamin D and the placebo group were performed using unpaired Student’s *t* test, Mann–Whitney *U* test, *X*^2^ test, or Fisher’s exact test, as appropriate

*25(OH)D* 25-hydroxyvitamin D, *AUCgluc* plasma glucose area under the curve, *BP* blood pressure, *CRP* C-reactive protein, *DHEAS* dehydroepiandrostendione-sulfate, *FAI* free androgen index, *FSH* follicle-stimulating hormone, *HbA1c* glycated hemoglobin, *HDL-cholesterol* high density lipoprotein-cholesterol, *HOMA-IR* homeostatic model assessment-insulin resistance, *LDL-cholesterol* low density lipoprotein-cholesterol, *LH* luteinizing hormone, *OGTT* glucose 30 min plasma glucose at 30 min during 75 g oral glucose tolerance test, *OGTT* glucose 60 min plasma glucose at 60 min during 75 g oral glucose tolerance test, *OGTT* glucose 120 min plasma glucose at 120 min during 75 g oral glucose tolerance test, *PTH* parathyroid hormone, *QUICKI* quantitative insulin sensitivity check index *WHR* waist-to-hip ratio

A total of 123 study participants [age 25.9 ± 4.7 years; BMI 27.5 ± 7.3 kg/m^2^; baseline 25(OH)D 48.8 ± 16.9 nmol/L; baseline fasting glucose 84 ± 8 mg/dL] completed both the baseline visit and the final follow-up visit after 24 weeks, while 140 participants [age 26.1 ± 4.8 years; BMI 27.5 ± 7.4 kg/m^2^; baseline 25(OH)D 48.1 ± 17.7 nmol/L] completed the baseline visit and the first follow-up visit after 12 weeks. The proportion of participants completing the study (i.e., study participation for 24 weeks) did not significantly differ between the vitamin D and placebo group (81 participants randomized to the vitamin D group and 42 participants to the placebo group; *p* = 1.00). The mean (± SD) overall treatment period was 176 ± 23 days in the vitamin D group and 176 ± 21 days in the placebo group (*p* = 0.906).

There was no significant effect of vitamin D supplementation on AUCgluc at study end (24 weeks) with a mean treatment effect [95% confidence interval (CI)] of − 9.19 (− 21.40 to 3.02, *p* = 0.139). Table 2 shows the effects of vitamin D supplementation on the predefined continuous secondary outcome parameters. Vitamin D supplementation lead to a significant decrease in plasma glucose after 60 min during OGTT, while it did not significantly affect any of the other continuous secondary outcome parameters (Table 2). At study end, 49.4% of the participants in the vitamin D group and 42.1% of the participants in the placebo group showed improved menstrual regularity when compared to the screening visit (*p* = 0.552). Regarding parameters of bone and mineral metabolism, vitamin D supplementation significantly increased serum concentrations of 25(OH)D and 1,25-dihydroxyvitamin D [1,25(OH)_2_D], while it significantly decreased serum levels of parathyroid hormone (PTH) (Table 3).

**Table 2:** Continuous secondary outcome variables at baseline and final follow-up at study end (24 weeks) in study participants with available values at both study visits

	Baseline	Follow-up (24 weeks)	Treatment effect (95% CI)	*p* value
Fasting glucose (mg/dL)				
Vitamin D (*n* = 81)	84 ± 8	82 ± 8	− 1.2 (− 3.6 to 1.3)	0.353
Placebo (*n* = 42)	84 ± 8	83 ± 7		
OGTT glucose 30 min (mg/dL)				
Vitamin D (*n* = 80)	133 ± 24	130 ± 23	− 1.6 (− 10.0 to 6.8)	0.711
Placebo (*n* = 42)	128 ± 25	129 ± 26		
OGTT glucose 60 min (mg/dL)				
Vitamin D (*n* = 80)	123 ± 39	105 ± 31	− 10.2 (− 20.2 to − 0.3)	0.045
Placebo (*n* = 42)	107 ± 31	107 ± 34		
OGTT glucose 120 min (mg/dL)				
Vitamin D (*n* = 81)	98 ± 24	88 ± 24	0.5 (− 7.6 to 8.6)	0.903
Placebo (*n* = 42)	93 ± 24	85 ± 24		
HbA1c (mmol/mol)^a^				
Vitamin D (*n* = 74)	33 (31–35)	33 (32–35)	− 0.4 (− 0.9 to 0.2)	0.192
Placebo (*n* = 38)	34 (32–35)	33 (32–35)		
HOMA-IR^a^				
Vitamin D (*n* = 81)	1.95 (1.09–3.51)	2.29 (1.43–3.47)	− 0.26 (− 0.80 to 0.27)	0.935
Placebo (*n* = 42)	2.15 (1.28–3.00)	2.31 (1.28–3.81)		
QUICKI^a^				
Vitamin D (*n* = 81)	0.345 (0.317−0.378)	0.337 (0.318−0.362)	− 0.004 (− 0.028 to 0.019)	0.823
Placebo (*n* = 42)	0.340 (0.324−0.367)	0.337 (0.317−0.368)		
Triglycerides (mg/dL)^a^				
Vitamin D (*n* = 79)	62 (49–85)	71 (52–93)	3 (− 7 to 12)	0.455
Placebo (*n* = 42)	78 (50–118)	74 (48–106)		
Total cholesterol (mg/dL)^a^				
Vitamin D (*n* = 79)	173 (158–188)	172 (158–189)	4 (− 3 to 11)	0.180
Placebo (*n* = 42)	179 (148–203)	172 (143–204)		
Total testosterone (mg/dL)^a^				
Vitamin D (*n* = 78)	1.60 (1.10–2.20)	1.55 (1.28–2.00)	0.09 (− 0.11 to 0.28)	0.616
Placebo (*n* = 41)	1.40 (1.15–1.80)	1.40 (1.20–1.90)		
Free testosterone (mg/dL)^a^				
Vitamin D (*n* = 77)	0.020 (0.016–0.032)	0.021 (0.015–0.029)	0.002 (− 0.002 to 0.005)	0.445
Placebo (*n* = 41)	0.019 (0.015–0.035)	0.021 (0.013–0.028)		

Data are shown as means with standard deviation or medians and interquartile range, as appropriate. Treatment effects with 95% confidence interval and *p* values were calculated by ANCOVA for group differences at follow-up with adjustment for baseline values

*HbA1c* glycated hemoglobin, *HOMA-IR* homeostatic model assessment-insulin resistance, *OGTT* glucose 30 min plasma glucose at 30 min during 75 g oral glucose tolerance test, *OGTT* glucose 60 min plasma glucose at 60 min during 75 g oral glucose tolerance test, *OGTT* glucose 120 min plasma glucose at 120 min during 75 g oral glucose tolerance test, *QUICKI* quantitative insulin sensitivity check index

^a^Skewed variables for which logarithmic transformed values were used in ANCOVA, but untransformed values are shown in the table

**Table 3:** Parameters of bone and mineral metabolism at baseline and final follow-up after 24 weeks in study participants with available values at both study visits

	Baseline	Follow-up (24 weeks)	Treatment effect (95% CI)	*p* value
25(OH)D (nmol/L)				
Vitamin D (*n* = 79)	48.8 ± 16.8	90.2 ± 20.1	33.4 (24.5 to 42.2)	< 0.001
Placebo (*n* = 41)	48.8 ± 17.5	56.8 ± 29.5		
PTH (pg/mL)*				
Vitamin D (*n* = 81)	41.9 (34.4–53.8)	40.6 (32.4–51.1)	− 6.6 (− 11.3 to − 1.9)	0.004
Placebo (*n* = 42)	40.2 (33.0-51.4)	45.7 (37.6–55.5)		
1,25(OH)_2_D (pmol/L)				
Vitamin D (*n* = 75)	114 ± 48	141 ± 52	27 (8 to 46)	0.006
Placebo (*n* = 41)	110 ± 43	113 ± 48		
Plasma calcium (mmol/L)				
Vitamin D (*n* = 79)	2.35 ± 0.08	2.32 ± 0.07	0.02 (− 0.003 to 0.05)	0.081
Placebo (*n* = 41)	2.36 ± 0.07	2.32 ± 0.07		

Data are shown as means with standard deviation or medians and interquartile range, as appropriate. Treatment effects with 95% confidence interval and *p* values were calculated by ANCOVA for group differences at follow-up with adjustment for baseline values

*1,25(OH)*_*2*_*D* 1,25-dihydroxy vitamin D, *25(OH)D* 25-hydroxyvitamin D, *PTH* parathyroid hormone

^a^Skewed variables for which logarithmic transformed values were used in ANCOVA, but untransformed values are shown in the table

Effects of vitamin D supplementation on primary and secondary outcome parameters after 12 weeks are shown in Supplemental Table 2. In accordance with the results after 24 weeks, vitamin D supplementation significantly reduced plasma glucose after 60 min during the oral glucose tolerance test. Further, we observed a significant decrease in AUCgluc after 12 weeks (Supplemental Table 2).

In subgroup analyses among participants with a baseline 25(OH)D concentration < 50 nmol/L (*n* = 60), vitamin D supplementation significantly reduced AUCgluc after 24 weeks with a mean treatment effect (95% CI) of − 19.20 (− 35.45 to − 2.95, *p* = 0.021). Regarding secondary outcome parameters, we found a significant decrease in plasma glucose after 60 min during OGTT (mean treatment effect − 17.8 mg/dL; 95% CI − 31.0 to − 4.5; *p* = 0.010) whereas no significant change was found for the remaining secondary outcome parameters. In subgroup analyses in PCOS patients with baseline 25(OH)D concentrations < 40 nmol/L (*n* = 39), we observed no significant effects of vitamin D supplementation on either primary or secondary outcome parameters (data not shown).

No unintended treatment effects or serious adverse events were observed during the study. No study participant treated with vitamin D developed hypercalcemia at either of the follow-up visits.

## Discussion

In this RCT in women with PCOS and 25(OH)D serum concentrations below 75 nmol/L, we found no significant effect of vitamin D supplementation on AUCgluc (primary outcome) or on other metabolic and endocrine parameters, with the exception of a significant decrease in plasma glucose after 60 min during OGTT. Furthermore, we were unable to observe a significant improvement in menstrual frequency in the vitamin D group at study end.

The pathophysiological backgrounds of possible vitamin D effects in PCOS are still not fully elucidated. In PCOS, the associations between vitamin D deficiency and insulin resistance do not appear to be confounded by obesity [28]. An alternative hypothesis suggests that vitamin D may stimulate the expression of insulin receptors and improve the insulin responsiveness for glucose transport, since 1,25(OH)_2_D leads to transcription activation of the insulin gene while the vitamin D responsive element is present in the promotor region of the human insulin gene [13, 29, 30]. Possible impacts of vitamin D status on androgen levels, menstrual frequency, or fertility may be explained by the ubiquitous expression of the vitamin D receptor within the female reproduction system [13–15]. 1,25(OH)_2_D furthermore directly leads to the production of estrogen and progesterone in cultured human ovary and placenta cells [15, 31], thereby possibly leading to an improved endometrial environment by potentiating granulosa cell luteinization [12].

In the recent past, several other RCTs have reported diverse results regarding the effect of vitamin D supplementation in PCOS, leaving the role of vitamin D in the treatment of the syndrome unclear. Some authors found significant effects on key features of PCOS, such as Jamilian et al. [32], who investigated the effect of 4000 IU of cholecalciferol daily vs. 1000 IU of calciferol daily vs. placebo over 12 weeks in 90 PCOS women. They reported a significant decrease in fasting plasma glucose, serum insulin, HOMA-IR, TT, FAI, and hirsutism as well as a significant increase in SHBG and total antioxidant capacity in the group receiving 4000 IU of cholecalciferol daily. Likewise, Maktabi et al. [33] observed a significant decrease in fasting plasma glucose, HOMA-IR, homeostasis model assessment-estimated beta cell function (HOMA-β), CRP, and plasma malondialdehyde in 70 PCOS women receiving 50,000 IU of cholecalciferol every 14 days vs. placebo over 12 weeks. Other RCTs, however, were unable to find significant effects of vitamin D supplementation in PCOS: Raja-Khan et al. [34] found no significant effect of 12,000 IU of cholecalciferol daily vs. placebo over 12 weeks on QUICKI or other measures of insulin sensitivity in 28 PCOS women. Similarly, Garg et al. [35] reported no significant differences in several measures of insulin resistance and sensitivity in 36 PCOS women receiving either 120,000 IU of cholecalciferol monthly vs. placebo over 6 months (subjects in both groups additionally received 1500 mg of metformin daily).

The inconsistency in the current literature may be, at least in part, explained by the differences in study designs as well as the broad variation in study population sizes. Additionally, the dosing regimens of vitamin D showed differences in regard to quantity and frequency (e.g., daily vs. weekly vs. monthly supplementation), therefore, also potentially affecting the comparability of some studies.

In line with some of the aforementioned trials as well as a recent meta-analysis [17], our data do not suggest a significant impact of vitamin D supplementation on key features of the PCOS phenotype. The clinical relevance of our finding of decreased plasma glucose after 60 min during OGTT remains unclear, especially since there was no significant effect of vitamin D supplementation on our primary outcome measure (AUCgluc). Furthermore, we are well aware of the fact that multiple testing of various parameters of glucose metabolism increases the probability of statistical type 1 errors. The same appears to be true for our results concerning the first follow-up visit after 12 weeks: since our study was powered to detect treatment differences after 24 weeks, these results should be interpreted with caution. Furthermore, subgroup analyses in individuals with particularly low 25(OH)D concentrations at baseline (i.e., < 50 nmol/L) showed a possible effect of vitamin D supplementation on AUCgluc. These post hoc analyses possess serious general caveats [36] and were performed in a numerically significantly decreased study population. Thus, these results must be interpreted with extreme caution. However, we cannot rule out that vitamin D supplementation in individuals with lower baseline 25(OH)D concentrations might have a significant effect on our primary study outcome.

Our study has several limitations and strengths that need discussion. A possible limitation is the relatively high drop-out rate that made a total enrollment of 180 study participants necessary to meet the required numbers generated by our sample size calculations. As depicted in the participant flow-chart (Fig. 1), the major drop-out reason was unwillingness to continue the study due to e.g. no interest in further study participation, preference to use hormonal contraception, or unplanned stays abroad, while some subjects had to be excluded due to unplanned pregnancies during study participation. Nevertheless, it should be noted that in spite of the drop-out rate, the study population size of our trial is still very high in comparison to similar RCTs. Our findings should also be interpreted in light of potential limitations due to multiple testing as we analyzed eight different measures of glucose metabolism at different study visits. Another limitation may be the relatively high baseline concentrations of 25(OH)D, which was chosen according to published guidelines [19]. Therefore, we cannot rule out that vitamin D supplementation in PCOS women with lower concentrations might lead to different results. Since participants were recruited regardless of the presence of insulin resistance, we also cannot rule out a possible effect of vitamin D supplementation in a cohort of PCOS patients with insulin resistance. As an Austrian monocentric study, our results may not be generalizable to other populations within or outside of Europe.

A strength of our study is its design to specifically detect vitamin D effects on glucose response in PCOS women. Furthermore, we used a state of the art method to measure concentrations of 25(OH)D and TT. To the best of our knowledge, this is the first RCT investigating the effects of vitamin D in PCOS using ID–LC–MS/MS to measure both of these parameters. The validity of our data is underscored by confirmation of the well-known treatment effects of vitamin D supplementation on 25(OH)D, PTH, and 1,25(OH)_2_D [37, 38].

In conclusion, we did not find significant effects of vitamin D supplementation on either metabolic or endocrine parameters in our cohort of PCOS women with insufficient baseline 25(OH)D concentrations, except for a decrease in plasma glucose after 60 min during OGTT. These results need to be confirmed in other cohorts with comparable or even larger population sizes.

## Electronic supplementary material

Below is the link to the electronic supplementary material.

Supplementary material 1 (PDF 276 KB)
