# Supplementary material for: Effects of vitamin D supplementation on metabolic and endocrine parameters in PCOS: a randomized-controlled trial
Source: Eur J Nutr. 2018 Jun 26;58(5):2019–28. doi: 10.1007/s00394-018-1760-8 (PMC6647224; doi:10.1007/s00394-018-1760-8)
Supplement: Supplementary file 1 — Supplementary material 1 (PDF 276 KB) [file 394_2018_1760_MOESM1_ESM.pdf]

Effects of vitamin D supplementation on metabolic and endocrine parameters in PCOS: a randomized-controlled trial

Christian Trummer*, Verena Schwetz, Martina Kollmann, Monika Wölfler, Julia Münzker, Thomas R. Pieber, Stefan Pilz, Annemieke C. Heijboer, Barbara Obermayer-Pietsch, Elisabeth Lerchbaum

*Division of Endocrinology and Diabetology, Department of Internal Medicine, Medical University of Graz, Auenbruggerplatz 15, 8036 Graz, Austria; christian.trummer@medunigraz.at

European Journal of Nutrition

Supplemental Materials and Methods

Procedures

Anthropometric measurements (height, weight, waist and hip circumference, blood pressure) were obtained from all subjects at each study visit. Blood pressure was measured after a resting period of at least five minutes. Body-mass index (BMI) was calculated as body weight [kg] divided by the height in meters squared. To evaluate the presence of clinical hyperandrogenism for the diagnosis of PCOS, hirsutism was classified according to the modified Ferriman-Gallwey score [1].

Mass spectrometry (MS) measurements

Measurements of 25(OH)D and TT by ID-LC-MS/MS were performed at the Endocrine Laboratory of the VU University Medical Center, Amsterdam, the Netherlands, as previously described [2,3].

For 25(OH)D, the internal standards $^{13}C_5$–25(OH)D_3 and $^{2}H_6$–25(OH)D were added to the samples and 25(OH)D was released from its binding proteins with acetonitrile. After a liquid-liquid extraction by hexane, the samples were analyzed by LC-MS/MS (Acquity UPLC coupled to a Quattro Premier XE MS/MS, Waters Corp., Milford, MA, USA). For concentrations between 17 and 160

nmol/L, intraassay and interassay coefficients of variation (CVs) were <4.5% and <5.5%, respectively. The limit of quantification (LOQ) was 4.0 nmol/L.

For TT, the internal standard $^{13}C_3$-testosterone was added to the samples and testosterone was released from its binding proteins with acetonitrile. After a liquid-liquid extraction by hexane, the samples were analyzed by LC-MS/MS (Acquity 2D-UPLC coupled to a Xevo TQ-S tandem mass spectrometer, Waters Corp., Milford, MA, USA). Interassay variation at 0.1 nmol/L was 10.6% and <6% between 0.9 and 14 nmol/L. The LOQ was 0.10 nmol/L.

Both methods to measure either 25(OH)D and TT were well-standardized [2,4].

Biochemical analyses

To evaluate inclusion criteria, 25(OH)D was initially measured by a commercially available enzyme immunoassay (IDS, Boldon, UK) with intra- and interassay CVs of 5.6 and 6.4%, respectively. To establish the diagnosis of PCOS, TT was initially measured by luminescence immunoassay (Siemens, Erlangen, Germany) with intra- and interassay CVs of <10%. Insulin was measured by luminescence immunoassay (Siemens, Erlangen, Germany) with intra- and interassay CVs of 4% and 2.6%, respectively. PTH was measured by ElectroChemiLuminescence immunoassay (ECLIA; Roche Diagnostics, Mannheim, Germany) with intra- and interassay CVs of 1.5-2.7% and 3.0-6.5%, respectively. Sex hormone-binding globulin (SHBG; for calculation of FT and FAI) was measured by luminescence immunoassay (Cobas, Roche, Basel, Switzerland) with intra- and interassay CVs of 1.3% and 2.1%, respectively. Androstendione and dehydroepiandrostendione-sulfate (DHEAS) were measured by ELISA (DiaMetra, BioVendor, Brno, Czech Republic and LDN Labor Diagnostika Nord GmbH, Nordhorn, Germany) with intra- and interassay CVs of <10%. Estradiol was measured by chemiluminescence immunoassay (Siemens, Erlangen, Germany) with intra- and interassay CVs of 6.3-15% and 6.4-16%, respectively. Follicle-stimulating hormone (FSH) and luteinizing hormone (LH) were measured by enzyme immunoassay (DiaMetra S.r.l., Segrate (MI), Italy) with intra- and interassay CVs of <10%. 1,25$(OH)_2$D was measured by chemiluminescence immunoassay (IDS, Boldon, UK) with intra- and interassay CVs of 6.4-12.1% and 6.6-9.6%, respectively.

All other parameters [plasma glucose, HbA1c, triglycerides, TC, HDL-cholesterol, LDL-cholesterol, C-reactive protein (CRP), plasma calcium] were determined by routine laboratory diagnostics. To avoid glycolysis and therefore incorrect measurements, plasma glucose was measured from tubes containing sodium fluoride.

Supplemental Table 1: Normal ranges of baseline biochemical and anthropometric parameters (where available) and participants within these ranges

Parameter	Normal Range	Participants [%]
Body-mass index (kg/m^2)	<18.5 underweight<br>18.5-25.0 normal weight<br>25.0-30.0 overweight<br>>30.0 obese	0.8<br>49.2<br>19.7<br>30.3
Waist circumference (cm) [5]	<80	32.1
WHR (cm/cm) [6]	<0.85	54.2
Systolic BP (mmHg) [7]	<130	79.2
Diastolic BP (mmHg) [7]	<85	70.8
Fasting glucose (mg/dL) [8]	<100	98.4
OGTT glucose 120 min (mg/dL) [8]	<140 (for prediabetes)<br><200 (for diabetes)	95.1<br>100
Fasting insulin (mU/L)	3.0-25.0	85.4
HbA1c (mmol/mol) [8]	<39 (for prediabetes)<br><48 (for diabetes)	91.9<br>100
Triglycerides (mg/dL)	<150	91.7
Total cholesterol (mg/dL)	<200	78.5
HDL-cholesterol (mg/dL)	>40	92.5
CRP (mg/L)	<5	84.9
25(OH)D (nmol/L)*	75-150	0
PTH (pg/mL)	15.0-65.0	89.4
Plasma calcium (mmol/L)	2.20-2.65	95.1
Total testosterone (nmol/L)[+] [9]	0.30-1.69	60.2
Androstendione (ng/mL)	0.75-3.20	47.2
DHEAS (µg/mL)	0.46-2.75	73.2

*Normal range of the commercially available immunoassays used to evaluate study eligibility. [+]Normal range of the ID-LC-MS/MS method used to measure total testosterone; for recruitment and to establish the clinical diagnosis of polycystic ovary syndrome, a commercially available immunoassay measurement was used

25(OH)D = 25-hydroxyvitamin D; BP = blood pressure; CRP = C-reactive protein; DHEAS = dehydroepiandrostendione-sulfate; HbA1c = glycated hemoglobin; HDL-cholesterol = high density lipoprotein-cholesterol; LDL-cholesterol = low density lipoprotein-cholesterol; OGTT glucose 120 min = plasma glucose at 120 minutes during 75g oral glucose tolerance test; PTH = parathyroid hormone; WHR = waist-to-hip ratio

Supplemental Table 2: Primary and secondary outcome variables at baseline and final follow-up after 12 weeks in study participants with available values at both study visits

	Baseline	Follow-Up (12 weeks)	Treatment Effect (95% CI)	p-value
AUCgluc				
Vitamin D (n=92)	228.05±46.45	210.59±46.35	-12.89 (-24.70 to -1.08)	0.033
Placebo (n=45)	210.04±40.39	209.77±47.54		
Fasting glucose [mg/dL]				
Vitamin D (n=94)	84±8	83±9	-0.5 (-2.9 to 1.9)	0.681
Placebo (n=46)	84±8	84±7		
OGTT glucose 30 min [mg/dL]				
Vitamin D (n=91)	133±25	131±29	-3.0 (-11.0 to 4.9)	0.452
Placebo (n=45)	127±24	130±23		
OGTT glucose 60 min [mg/dL]				
Vitamin D (n=91)	122±40	108±35	-10.8 (-20.0 to -1.6)	0.022
Placebo (n=45)	107±31	108±39		
OGTT glucose 120 min [mg/dL]				
Vitamin D (n=92)	99±24	87±21	-2.8 (-9.9 to 4.3)	0.430
Placebo (n=45)	91±24	86±25		
*HbA1c [mmol/mol]**				
Vitamin D (n=42)	33 (31-35)	34 (32-35)	0.4 (-0.2 to 1.0)	0.166
Placebo (n=85)	34 (32-35)	33 (32-35)		
*HOMA-IR**				
Vitamin D (n=94)	1.92 (1.09-3.49)	2.32 (1.34-3.62)	0.52 (-0.96 to 1.99)	0.134
Placebo (n=46)	2.15 (1.28-3.00)	2.02 (1.06-3.23)		
*QUICKI**				
Vitamin D (n=94)	0.346 (0.318-0.378)	0.336 (0.316-0.366)	-0.009 (-0.022 to 0.003)	0.129
Placebo (n=46)	0.340 (0.324-0.369)	0.343 (0.321-0.380)		
*Triglycerides [mg/dL]**				
Vitamin D (n=91)	63 (49-90)	69 (53-94)	5 (-4 to 15)	0.455
Placebo (n=45)	74 (51-114)	76 (58-95)		
*Total cholesterol [mg/dL]**				
Vitamin D (n=91)	172 (156-190)	179 (151-194)	0.3 (-7 to 7)	0.780
Placebo (n=45)	175 (142-203)	172 (148-197)		
*Total testosterone [nmol/L]**				
Vitamin D (n=92)	1.55 (1.13-2.18)	1.50 (1.10-2.00)	-0.14 (-0.34 to 0.06)	0.164
Placebo (n=44)	1.40 (1.20-1.80)	1.60 (1.13-1.88)		
*Free testosterone [nmol/L]**				
Vitamin D (n=91)	0.021 (0.016-0.032)	0.021 (0.014-0.029)	-0.001 (-0.004 to 0.002)	0.271
Placebo (n=44)	0.019 (0.015-0.035)	0.022 (0.015-0.031)		

Data are shown as means with standard deviation or medians and interquartile range, as appropriate. Treatment effects with 95% confidence interval and p-values were calculated by ANCOVA for group differences at follow-up with adjustment for baseline values.

*Skewed variables for which logarithmic transformed values were used in ANCOVA, but untransformed values are shown in the table.

AUCgluc = plasma glucose area under the curve; HbA1c = glycated hemoglobin; HOMA-IR = homeostatic model assessment-insulin resistance; OGTT glucose 30 min = plasma glucose at 30 minutes during 75g oral glucose tolerance test; OGTT glucose 60 min = plasma glucose at 60 minutes during 75g oral glucose tolerance test; OGTT glucose 120 min = plasma glucose at 120 minutes during 75g oral glucose tolerance test; QUICKI = quantitative insulin sensitivity check index

References

1. Yildiz BO, Bolour S, Woods K, Moore A, Azziz R (2010) Visually scoring hirsutism. Hum Reprod Update 16:51–64. https://doi.org/10.1093/humupd/dmp024
2. Dirks NF, Vesper HW, van Herwaarden AE, van den Ouweland JM, Kema IP, Krabbe JG, Heijboer AC (2016) Various calibration procedures result in optimal standardization of routinely used 25(OH)D ID-LC-MS/MS methods. Clin Chim Acta 462:49–54. https://doi.org/10.1016/j.cca.2016.08.016
3. Büttler RM, Martens F, Kushnir MM, Ackermans MT, Blankenstein MA, Heijboer AC (2015) Simultaneous measurement of testosterone, androstenedione and dehydroepiandrosterone (DHEA) in serum and plasma using isotope-dilution 2-dimension ultra high performance liquid-chromatography tandem mass spectrometry (ID-LC-MS/MS). Clin Chim Acta 438:157–159. https://doi.org/10.1016/j.cca.2014.08.023
4. Büttler RM, Martens F, Fanelli F, Pham HT, Kushnir MM, Janssen MJ, Owen L, Taylor AE, Soeborg T, Blankenstein MA, Heijboer AC (2015) Comparison of 7 published LC-MS/MS methods for the simultaneous measurement of testosterone, androstenedione, and dehydroepiandrosterone in serum. Clin Chem 61:1475–1483. https://doi.org/10.1373/clinchem.2015.242859
5. Lean ME, Han TS, Morrison CE (1995) Waist circumference as a measure for indicating need for weight management. BMJ 311:158-161. https://doi.org/10.1136/bmj.311.6998.158
6. World Health Organization (2011) Waist circumference and waist-hip ratio: report of a WHO expert consultation. World Health Organization, Geneva
7. Mancia G, Fagard R, Narkiewicz K, Redon J, Zanchetti A, Böhm M, Christiaens T, Cifkova R, De Backer G, Dominiczak A, Galderisi M, Grobbee DE, Jaarsma T, Kirchhof P, Kjeldsen SE, Laurent S, Manolis AJ, Nilsson PM, Ruilope LM, Schmieder RE, Sirnes PA, Sleight P, Viigimaa M, Waeber B, Zannad F, Redon J, Dominiczak A, Narkiewicz K, Nilsson PM, Burnier M, Viigimaa M, Ambrosioni E, Caufield M, Coca A, Olsen MH, Schmieder RE, Tsioufis C, van de Borne P, Zamorano JL, Achenbach S, Baumgartner H, Bax JJ, Bueno H, Dean V, Deaton C, Erol C, Fagard R, Ferrari R, Hasdai D, Hoes AW, Kirchhof P, Knuuti

J, Kolh P, Lancellotti P, Linhart A, Nihoyannopoulos P, Piepoli MF, Ponikowski P, Sirnes PA, Tamargo JL, Tendera M, Torbicki A, Wijns W, Windecker S, Clement DL, Coca A, Gillebert TC, Tendera M, Rosei EA, Ambrosioni E, Anker SD, Bauersachs J, Hitij JB, Caulfield M, De Buyzere M, De Geest S, Derumeaux GA, Erdine S, Farsang C, Funck-Brentano C, Gerc V, Germano G, Gielen S, Haller H, Hoes AW, Jordan J, Kahan T, Komajda M, Lovic D, Mahrholdt H, Olsen MH, Ostergren J, Parati G, Perk J, Polonia J, Popescu BA, Reiner Z, Rydén L, Sirenko Y, Stanton A, Struijker-Boudier H, Tsioufis C, van de Borne P, Vlachopoulos C, Volpe M, Wood DA (2013) ESH/ESC guidelines for the management of arterial hypertension: the Task Force for the Management of Arterial Hypertension of the European Society of Hypertension (ESH) and the European Society of Cardiology (ESC). Eur Heart J 34:2159-2219. https://doi.org/10.1093/eurheartj/eht151

8. American Diabetes Association (2018) 2. Classification and Diagnosis of Diabetes: Standards of Medical Care in Diabetes-2018. Diabetes Care 41:S13-S27. https://doi.org/10.2337/dc18-S002

9. Bui HN, Sluss PM, Blincko S, Knol DL, Blankenstein MA, Heijboer AC (2013) Dynamics of serum testosterone during the menstrual cycle evaluated by daily measurements with an ID-LC-MS/MS method and a 2nd generation automated immunoassay. Steroids 78:96-101. https://doi.org/10.1016/j.steroids.2012.10.010
